# PERMA-based mobile psychological intervention with and without telephone support for university students: study protocol for a randomized controlled trial

**DOI:** 10.3389/fpsyt.2026.1854387

**Published:** 2026-07-09

**Authors:** Vanessa Blanco, Fataneh Sinaeipour, Queila Bouza, Fernando L. Vázquez

**Affiliations:** 1 Department of Evolutionary and Educational Psychology, University of Santiago de Compostela, Santiago de Compostela, Spain; 2 Department of Clinical Psychology and Psychobiology, University of Santiago de Compostela, Santiago de Compostela, Spain

**Keywords:** mobile intervention, PERMA model, psychological well-being, randomized controlled trial, university students

## Abstract

**Background:**

University students face increasing academic, developmental, and psychosocial demands that place them at risk of reduced psychological well-being. Although positive psychology interventions have shown promising effects in this population, accessibility to them is limited. This barrier can be overcome by employing new technologies. However, none of the existing interventions that used ICT in this population was based on Seligman´s PERMA model, and they report low adherence rates. Complementing mobile-based interventions with brief telephone support may improve engagement and enhance outcomes. This study aims to evaluate the efficacy of a positive psychology–based psychological intervention grounded in the PERMA model delivered via a smartphone app, incorporating cognitive-behavioral self-regulation strategies (e.g., goal setting, self-monitoring, behavioral activation), with or without telephone multiconference support.

**Methods:**

A three-arm randomized controlled trial will be conducted with 177 university students, who will be assigned to: (a) an app-based PERMA intervention (PPIA), (b) the same intervention supplemented with weekly 30-minute telephone multiconference sessions (PPIA+M), or (c) a waiting list control group (WLCG). The intervention consists of five weekly modules targeting Positive Emotion, Engagement, Relationships, Meaning, and Accomplishment. Assessments will be conducted at baseline, post-intervention, and six-month follow-up. The primary outcome will be psychological well-being, measured with the Warwick–Edinburgh Mental Well-Being Scale (WEMWBS) and the Mental Health Continuum–Short Form (MHC-SF). Secondary outcomes include positive emotion, engagement, relationships, meaning, accomplishment, adherence, and satisfaction. Evaluations will be administered by independent, blinded assessors.

**Discussion:**

This study will provide evidence on the efficacy and acceptability of a brief PERMA-based digital intervention for university students. If effective, the program may represent a scalable and cost-efficient tool for promoting psychological well-being in higher education settings.

**Study Protocol Registration:**

ClinicalTrials.gov, identifier (NCT07466004).

## 1 Introduction

Young adulthood, which generally spans from late teenage years to the mid-20s, represents a crucial period in people´s lives, marked by significant transitions that shape their future (1–3). During this phase, young adults face various challenges, such as pursuing higher education, establishing independence, forming intimate relationships, and making career decisions (4). These tasks are crucial for acquiring autonomy, exploring personal identity, and finding purpose, all of which are key elements of positive youth development (5, 6). Furthermore, the phase of young adulthood is characterized by individuals progressively assuming adult responsibilities, such as achieving financial autonomy, developing personal values, and taking charge of their health and wellness (7). Accomplishing these developmental tasks contributes to overall well-being, laying the foundation for future academic and career achievements, fulfilling relationships, and overall life satisfaction. Within the broader tapestry of society, university students emerge as a particularly significant demographic (8). They are not merely a subset of the population; they form a considerable proportion of their age group (9). In addition to these evolutionary achievements, university students face multiple academic and psychosocial demands that can deteriorate their psychological well-being and place them at risk for negative consequences on their mental health and academic functioning (10). The high prevalence of mental health problems such as anxiety and depression among university students has been well-documented in the literature (11, 12). These problems are associated with decreased academic performance, lower retention rates, and increased risk of dropout (13).

For all these reasons, the promotion of mental health in this population sector acquires vital relevance. Research findings confirm that prioritizing positive mental health enhances academic performance (14). Additionally, positive psychological well-being correlates strongly with improved interpersonal relationships and social integration within the university community. Students who manifest a state of positive mental health are characterized by robust communication abilities, elevated self-regard, and amplified empathetic responses; this constellation of attributes culminates in the cultivation of more fulfilling interpersonal connections with their contemporaries, academic personnel, and administrative staff (15). Furthermore, maintaining a positive state of mental health is essential for overall well-being and life quality; individuals who experience good mental health often report increased levels of life satisfaction, a heightened sense of purpose, and greater overall happiness (16, 17).

In recent times, the significance and recognition of mental health promotion have notably increased, especially as a crucial method to bolster the mental and emotional health of university students. As outlined by the National Research Council & Institute of Medicine (18), it involves strategies aimed at improving developmental competencies, self-esteem, mastery, well-being, and social inclusivity, while also enhancing resilience against challenges. It prioritizes empowering individuals and shaping environments that nurture mental health, going beyond merely addressing mental illness. The approach integrates proactive steps to fortify protective factors and foster positive mental health outcomes. It comprehensively considers the intricate aspects of psychological well-being, influenced by diverse social, cultural, and environmental elements. By leveraging techniques that underscore strengths, resilience, and affirmative relationships, it seeks to bolster both personal and communal resources, ultimately aspiring to elevate mental health and overall life quality (19).

Over the past several years, several studies have applied Seligman’s PERMA model (20)—which includes Positive Emotion, Engagement, Relationships, Meaning, and Accomplishment—in university contexts to enhance psychological well-being. The interventions based on this theoretical approach emphasize building students’ strengths and emotional resources through practical, reflective activities tied to everyday experiences. Among them, Sedeh and Aghaei (21) conducted a six-session program for university students in Iran, with each session focusing on one PERMA component. Activities included gratitude journaling, exploring personal strengths, group discussions, values clarification, and goal planning, resulting in significant improvements in psychological well-being. Similarly, Yang et al. (22) implemented a PERMA-based intervention with vocational nursing students in China to reduce social interaction anxiety and enhance subjective well-being. The program included mindfulness practice, journaling, empathy-building tasks, and goal tracking, and showed notable improvements in both emotional and social functioning. In a language learning context, Rogers et al. (23) integrated PERMA principles into English instruction. Weekly sessions featured activities like “three good things” journaling, peer appreciation, and reflections on meaningful experiences. Students reported increased engagement and connection, suggesting that PERMA-based activities can effectively enhance classroom dynamics. Finally, Ly and Nguyen (24) introduced PERMA-informed lessons in English-speaking classes, using storytelling, emotional vocabulary exercises, and reflective journaling, which helped students improve both language performance and emotional awareness. These findings support the long-term benefits of building strengths across these five areas. However, despite the promising outcomes associated with PERMA-based interventions, issues of accessibility remain a significant challenge. Many university students still face barriers to participation due to stigma, lack of time, or insufficient availability of mental health resources (25). To address these challenges, the integration of digital tools—such as mobile applications—has been increasingly explored as a scalable and flexible solution (26, 27).

A growing body of research has demonstrated the efficacy of interventions rooted in positive psychology for promoting mental health and well-being among young adults, particularly when delivered through mobile applications. For example, Bendtsen et al. (28) conducted a randomized controlled trial evaluating the impact of a positive psychology–based intervention delivered via a mobile health (mHealth) application. Compared to a control group that received only online mental health information, the intervention group showed significant improvements in positive mental health and reductions in anxiety and depression symptoms at the six-month follow-up. Similarly, Mak et al. (29) compared the effectiveness of basic mindfulness and Health Action Process Approach (HAPA)-enhanced mindfulness programs with a waitlist control group. Both intervention groups demonstrated significant improvements in psychological well-being, which were sustained at the three-month follow-up. However, it´s important to note that none of the reviewed interventions was based on the PERMA model (20). Moreover, a key issue identified in both studies is low adherence rates; Bendtsen et al. (28) reported that participants completed an average of only 2.8 out of 6 planned sessions, highlighting a significant adherence challenge; similarly, Mak et al. (29) noted that just 55% of participants completed the full three-month intervention.

Providing participants with regular check-ins and personalized support via phone could help foster stronger engagement and higher completion rates. Telephone calls offer a more personalized and emotionally engaging form of communication, facilitating deeper connections between users and mental health professionals or support networks. This human interaction may enhance motivation and user retention (30, 31). Furthermore, telephone calls can help bridge the digital divide by offering an alternative to fully digital platforms. For example, Martin et al. (32) found that participants receiving telephone support reported significantly lower psychological distress compared to those in self-administered intervention groups. Beintner and Jacobi (33) showed that consistent telephone prompts significantly increased adherence to an online aftercare program for women with bulimia nervosa. Similarly, Vázquez et al. (34) found that adding multiconference telephone contacts not only improved adherence but also enhanced the outcomes of an app designed to prevent depression in a sample of caregivers. Additionally, a systematic review by Baker et al. (35) supports the effectiveness of telephone-delivered psychosocial interventions in promoting adherence to treatment protocols among individuals with mental health conditions.

To our knowledge, this is the first randomized controlled trial to evaluate a fully PERMA-based psychological intervention delivered via a mobile application in university students, and to directly compare a self-guided app format with a blended format that incorporates synchronous group-based telephone support. By examining not only efficacy but also adherence, satisfaction, and mechanisms of change across PERMA dimensions, this study addresses critical gaps in the digital mental health literature regarding engagement and scalability of positive psychology interventions in higher education contexts. The primary objectives of this randomized controlled trial are to: (a) assess the efficacy of a positive psychology–based psychological intervention grounded in the PERMA model in promoting psychological well-being among university students administered through a smartphone app, with (PPIA + M) and without the addition of telephone multiconference calls (PPIA), compared to a waiting list control group (WLCG); and (b) examine the mediators and moderators of change in psychological well-being. The central hypotheses are that participants in both the PPIA and PPIA+M groups will demonstrate significantly greater improvements in psychological well-being compared to the WLCG at post-treatment and 6-month follow-up, with the PPIA+M group showing more pronounced improvements due to the added telephone support; additionally, it is expected that the change in positive emotion, engagement, relationships, meaning, and accomplishment will mediate the effects of both interventions; and that sociodemographic, academic, and clinical variables, as well as satisfaction with the intervention, will moderate treatment effects. Mediation and moderation analyses are planned as exploratory and hypothesis-generating, aimed at informing future optimization of the intervention.

## 2 Methods and analysis

### 2.1 Design

A randomized controlled trial (RCT) will be conducted. The present trial protocol follows the CONSORT (36) and SPIRIT (37) statements’ extensions for clinical trials, ensuring the inclusion of all key methodological aspects. Participants will be randomly assigned to one of three groups: (a) an app-based positive psychology–based psychological intervention grounded in the PERMA model (PPIA; experimental group 1); (b) an app-based positive psychology–based psychological intervention grounded in the PERMA model supplemented by telephone multiconference calls (PPIA+M; experimental group 2); or (c) a waiting list control group (WLCG).

**Figure 1:**
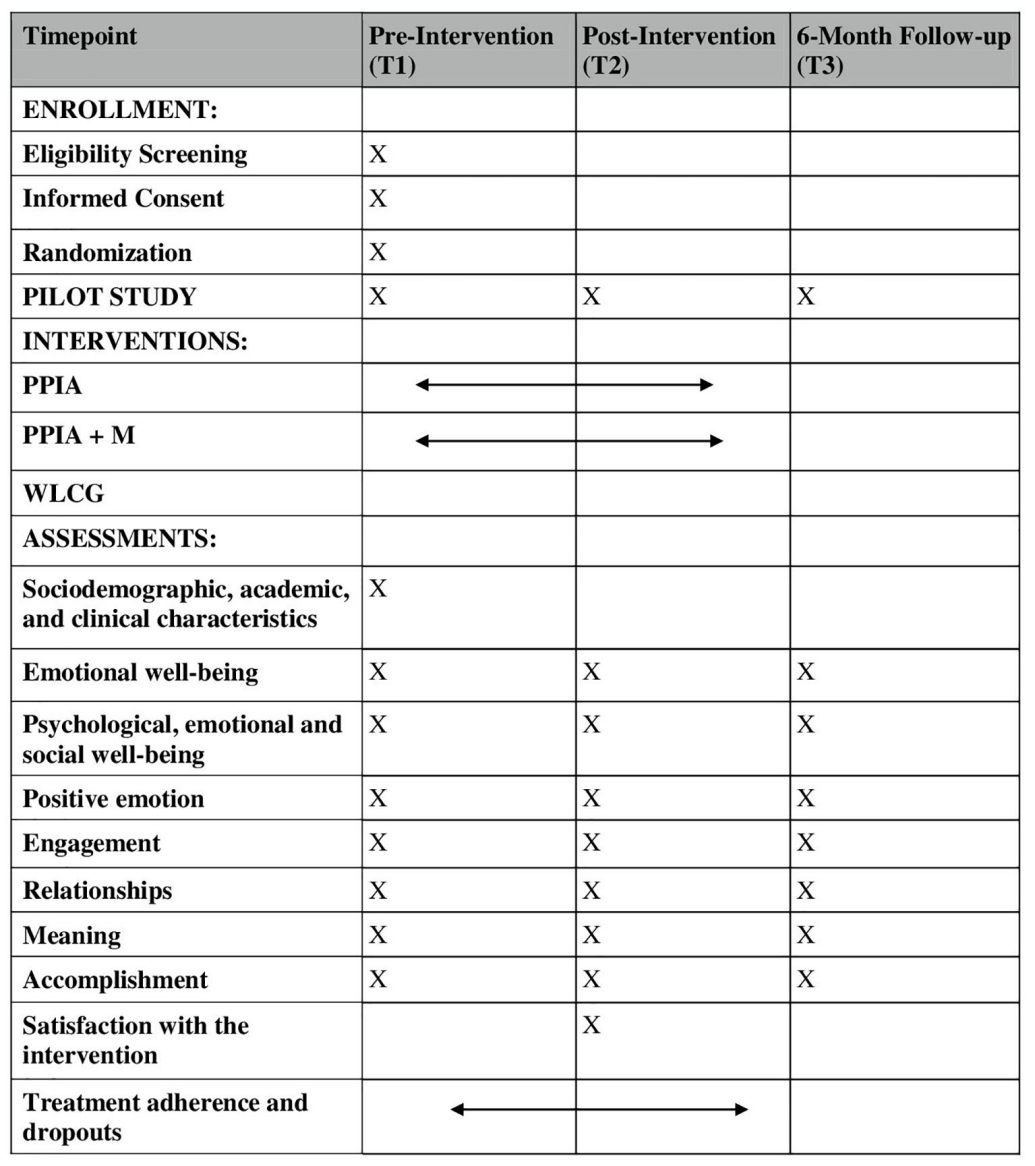
SPIRIT figure. Phases of a randomized controlled trial.

The study phases are illustrated in **Figure 1**, which depicts the flow of participants through the trial. Assessments will be conducted at three time points across the three groups: pre-intervention (T1), post-intervention (T2), and follow-up at 6 months (T3). After the baseline assessment, eligible participants will be randomized, and interventions will be administered over five weeks. The post-intervention assessment will be conducted immediately after completion, followed by a follow-up evaluation at 6 months. To ensure participant retention and limit attrition, strategies recommended in clinical research design will be employed. These include clear communication of expectations, structured reminder systems, brief follow-ups in case of missing data, and offering flexible scheduling for phone-based sessions in the PPIA+M group. The intervention app will be designed for ease of use to support consistent engagement (38).

### 2.2 Participants

#### 2.2.1 Recruitment

Participants will be recruited from the student population of the University of Santiago de Compostela (USC). According to the latest available data, USC enrolls approximately 24,095 students across its undergraduate, master’s, and doctoral programs. Recruitment will be conducted using multiple communication channels, including official USC digital platforms, targeted email invitations, printed posters in faculty buildings, and brief in-class announcements during psychology-related courses and student events.

Interested students will be contacted by phone to schedule the initial assessment session (T1) at the University of Santiago de Compostela’s facilities. During this in-person session, the study will be explained in detail, including information regarding the study’s objectives, procedures, potential risks, and expected benefits. Students who agree to participate will be asked to sign a written informed consent form. After obtaining consent, trained evaluators will collect sociodemographic, academic and clinical data and conduct a structured clinical interview (see Section 2.2.7.2.) to determine whether participants meet the eligibility criteria, specifically, the absence of severe psychiatric disorders. Individuals who meet all eligibility criteria will proceed with completing the remaining baseline assessment measures.

#### 2.2.2 Eligibility criteria

Inclusion criteria include: (a) being an actively enrolled university student at the University of Santiago de Compostela (USC); and (b) having access to a smartphone with an internet connection. Exclusion criteria include: (a) having a current diagnosis of severe psychiatric disorder, including major depressive disorder with psychotic features, bipolar disorder, schizophrenia, severe cognitive impairment, dissociative disorders, active substance dependence, or active suicidal behaviors; (b) having started psychological or psychopharmacological treatment within the two months prior to the study or participating in another mental health-related study; (c) having insufficient proficiency in Spanish, or presenting sensory, cognitive, or physical impairments that would prevent engagement with the intervention; or (d) planning to move out of Galicia within the next 12 months.

#### 2.2.3 Randomization

An independent researcher, who will not be involved in participant recruitment or data collection, will conduct the randomization to maintain allocation concealment. The researcher will generate the random allocation sequence using a computer-generated random number generator. The original randomization sequence will be securely stored by the independent researcher in an inaccessible location, and only the researcher will have access to the sequence. The sequence of assignments will be communicated to the researchers using sealed numbered envelopes, one for each participant, with instructions to open them in numerical order. Due to the nature of the interventions, participants will not be blinded to their treatment allocation.

#### 2.2.4 Sample size calculation

The sample size calculation was conducted using G*Power 3.1.9.7, selecting the F-test family and the “ANOVA: Repeated measures, within-between interaction” option. The calculation targeted the omnibus longitudinal group-by-time interaction in a design with three groups and three assessment points. The effect size was entered as Cohen’s *f* = 0.16, which represents a small effect and is approximately comparable to a standardized effect of *d/g* ≈ 0.32 under a simple two-group contrast approximation, based on prior meta-analytic findings that report small but consistent effects of app-based interventions on psychological well-being among university students (26). To account for the two co-primary outcomes, the alpha level was adjusted to α = .025 using a Bonferroni correction. The remaining parameters were statistical power = .95, correlation among repeated measures = .50, and nonsphericity correction *ϵ* = 1. Under these assumptions, the required analytical sample size was 141 participants, corresponding to 47 participants per group, with an actual power of .9509. To allow for an anticipated attrition rate of approximately 20%, the recruitment target was increased to 177 participants, corresponding to 59 participants per group.

#### 2.2.5 Ethics

The study will strictly adhere to established ethical standards, including the principles outlined in the Declaration of Helsinki (39) and the Ethical Principles of Psychologists and Code of Conduct of the American Psychological Association (40). In compliance with European and Spanish data protection regulations—specifically the General Data Protection Regulation (GDPR) (41) and the Spanish Organic Law 3/2018 on the Protection of Personal Data and guarantee of digital rights (42)—the study has been approved by the Research Ethics Committee of the University of Santiago de Compostela (Code: USC-003/2026-H). All personal data will be anonymized, securely stored, and accessible only to authorized personnel. Participation in the study will be entirely voluntary, without any financial or material incentives, and written informed consent will be obtained from all participants before involvement. Participants will be explicitly informed of their right to withdraw from the study at any time without penalty.

Suicide risk and acute distress will be assessed at each assessment point (T1, T2, and T3) using the Columbia–Suicide Severity Rating Scale Screen Version (C-SSRS Screen Version) (43). Positive responses indicating active suicidal behaviors will trigger the study safety protocol, including brief risk assessment by a trained psychologist, referral to the Psychological Care Unit of the University of Santiago de Compostela or emergency psychiatric services when needed, and exclusion or discontinuation from the intervention when acute risk is identified. The app will not collect diagnostic risk data or trigger automated risk alerts, but it will include a permanent “Help & Support” section with emergency contact information. Safety data and distress-related dropouts will be monitored monthly by the two senior clinical psychology supervisors. Adverse events will be documented and reported to the Research Ethics Committee.

Furthermore, participants assigned to the waiting list control group will be granted access to the app-based intervention after the study concludes. Any substantial modifications to the protocol will require prior approval from the relevant ethics committee before implementation.

#### 2.2.6 Interventions

A standardized, manualized intervention protocol will be developed for each experimental condition to ensure internal validity, consistency across delivery formats, and adherence to theoretical fidelity. The full intervention manual, including module outlines, facilitator guidance, fidelity and adherence checklists, and operational app-related materials, will be made available to independent researchers upon reasonable request once the trial is completed, subject to institutional and ethical restrictions.

Participants will be randomly assigned to one of three groups: (a) PPIA, a psychological intervention based on the PERMA model delivered via a mobile app; (b) PPIA+M, the same PERMA-based intervention delivered via app and supplemented with weekly telephone-based multiconference support sessions; and (c) WLCG (Waitlist Control Group), in which participants will not receive any intervention during the study period but will be granted access to the mobile intervention after completing all post-assessments. Although the intervention is grounded in positive psychology and the PERMA model, it integrates cognitive-behavioral principles such as structured goal setting, behavioral self-monitoring, action planning, and homework-based skill practice, which justify its classification as a CBT-informed intervention.

The two experimental conditions will consist of five modules, each corresponding to one of the five components of the PERMA model. Each module will last approximately 45 minutes and will be completed across one-week, engaging participants in three to five brief experiential and reflective activities per module (approximately 5–10 minutes per task). Content will be distributed across the week, and daily adherence will be encouraged through app notifications and visual progress tracking. In both intervention arms, task completion and engagement metrics will be automatically recorded by the app.

The intervention will be administered by licensed psychologists or doctoral students in clinical psychology with prior experience in digital and group interventions. The PPIA app will be entirely self-administered by participants, requiring no direct facilitation. In contrast, the multiconference sessions in the PPIA+M group will be administered by licensed psychologists or doctoral students in clinical psychology. These facilitators will undergo approximately 70 hours of structured training delivered by three senior clinical researchers with 20 to 35 years of experience in psychological therapies. The training will combine theoretical and practical seminars, a detailed review of intervention manuals, and extensive role-playing exercises to simulate delivery scenarios. The senior researchers responsible for the training will evaluate the facilitators’ adherence to the manuals and competence in group management, and will provide weekly supervision and feedback.

Following facilitator training and before conducting the randomized controlled trial, an internal pilot study will be carried out in which each facilitator will apply the intervention to approximately 15 participants. The objectives of the pilot are to review the acceptability and clarity of the intervention materials, evaluate the usability of the mobile application, and identify any procedural or technical challenges requiring refinement. Facilitator adherence to the manuals and delivery protocols will be assessed using structured observation checklists, and concordance of delivery and scoring among facilitators will be examined using Cohen’s kappa indices. In the PPIA+M arm, all multiconference sessions will be audio-recorded (with participant consent) and reviewed by the professionals responsible for facilitator training. These professionals will supervise facilitators weekly, evaluate their adherence to the manuals, assess their delivery skills, and provide corrective feedback where necessary.

Once the pilot data have been analyzed and refinements incorporated, recruitment for the full randomized controlled trial will commence following the procedures described in the Recruitment section. To maintain delivery quality and fidelity, facilitators will attend weekly 60-minute supervision meetings with the principal investigator and clinical coordinators. Supervision will involve case reviews, adherence monitoring, and problem-solving. Fidelity will be monitored using structured binary checklists assessing three core domains: adherence to PERMA content, competence in delivering Miltenberger-based feedback, and time management/group boundary control. A random sample of 20% of telephone sessions per facilitator will be independently rated by two raters using audio-recordings. Cohen’s kappa will be used to assess inter-rater agreement for the binary fidelity items. Acceptable fidelity will be defined as ≥80% adherence; if a facilitator falls below this threshold, targeted corrective supervision will be provided and the subsequent session will be monitored. Aggregated fidelity compliance scores and inter-rater reliability metrics will be reported in the final trial publication.

##### 2.2.6.1 PPIA – Positive psychology–based psychological intervention grounded in the PERMA model via App

The PPIA intervention is a structured psychological program grounded in the PERMA model of well-being (20). This model proposes that long-term flourishing depends on developing five pillars: Positive Emotion, Engagement, Relationships, Meaning, and Accomplishment. The intervention is further supported by the broaden-and-build theory of positive emotions (44), flow theory (45), research on character strengths and positive relationships (46, 47), meaning in life (48), the goal-setting theory (49) and growth mindset principles (50).

The program consists of five weekly modules, each lasting approximately 45 minutes and delivered through short daily sessions of 5–10 minutes. Each module includes psychoeducational content, experiential tasks, reflection exercises, and homework assignments designed to facilitate the integration of skills into daily life. Participants are expected to complete one module per week, with daily activities spread across the week (see **Table 1**). If a participant misses a module, the app allows delayed access so that it can be completed before advancing to the next, ensuring adherence to the sequence. The PPIA app is planned as a cross-platform mobile application compatible with iOS and Android devices. Core functions will include secure user authentication, encrypted data transmission through HTTPS/TLS protocols, sequential time-locked delivery of the five weekly modules, automated push notifications, visual progress tracking, and automatic recording of task completion and app engagement metrics. The app will not collect diagnostic risk data or generate automated clinical alerts.

**Table 1:** Contents, materials, and homework of the PERMA-based intervention.

Module	Session contents	Materials and homework
Module 1: Positive Emotion	Introduction to positive emotions; psychoeducation based on the broaden-and-build theory; awareness of positive daily experiences; practicing gratitude and savoring; emotional self-monitoring.	Materials: Short video/audio introduction; mood-tracking tool; gratitude journal template; savoring audio guide. Homework: “Three Good Things” exercise; daily gratitude entries; savoring one positive event; short reflection on emotional changes.
Module 2: Engagement	Understanding strengths vs. talents; identifying personal strengths (VIA framework); introduction to flow experiences; strategies to increase engagement in daily activities.	Materials: VIA strengths short inventory; flow diary template; strength-use instructions; daily progress visualizer. Homework: Complete VIA mini-test; record daily flow experiences; apply 1–2 personal strengths intentionally.
Module 3: Relationships	Psychoeducation on positive relationships; Active-Constructive Responding; empathy-building strategies; expressing appreciation; prosocial micro-actions.	Materials: Appreciation letter template; ACR practice scripts; empathy-walk guide; kindness task list. Homework: Write and optionally deliver an appreciation message; practice active-constructive responding; complete 2–3 micro-acts of kindness.
Module 4: Meaning	Exploring values and purpose; differences between happiness and meaning; identifying meaningful activities; connecting daily actions with personal values.	Materials: Life Compass worksheet; values clarification exercise; purpose-journaling prompts; meaning tracker. Homework: Daily intention-setting; 3-day purpose journal; future-self/legacy letter; one value-aligned action per day.
Module 5: Accomplishment	Goal-setting theory; developing self-efficacy; SMART goals; recognizing and reinforcing progress; planning achievable daily actions.	Materials: SMART goal planner; daily small-wins tracker; best-possible-self visualization; progress chart. Homework: Create weekly SMART goal; record daily small wins; complete Best Possible Self writing task; obstacle-planning reflection.

VIA, Values in Action; ACR, Active-Constructive Responding; SMART, Specific, Measurable, Achievable, Relevant, and Time-bound.

The app offers short-format tasks (5–10 minutes each) to be completed throughout the week. Each module includes a video or audio-based psychoeducational introduction (2–3 minutes), three to five experiential tasks with instructions, logging, and reflection, a personal journal to document insights and responses, interactive visualizations and self-monitoring tools (e.g., mood, progress), and daily motivational messages and reminders. In addition, participants will be assigned specific homework activities each week, such as keeping a gratitude journal, practicing mindfulness, or applying character strengths in daily life. These tasks are designed to consolidate learning, foster habit formation, and increase transfer of skills to real-world contexts.

In the first week, dedicated to Positive Emotion, the objective is to increase awareness, frequency, and intensity of positive affect in daily life. The psychoeducational component highlights the physiological, cognitive, and social benefits of cultivating positive emotions. Activities include *Three Good Things*, a daily gratitude journal organized by life domains, a savoring practice guided by mindfulness audio, and a positive visualization of a future joyful event, with supplemental mood tracking and a brief daily gratitude pause.

The second week, focused on Engagement, aims to help participants identify and apply their personal strengths to increase flow experiences. The module explains the distinction between talents and strengths and the benefits of using strengths regularly. Activities include completing an abbreviated *Values in Action (VIA) Character Strengths Inventory* with feedback, keeping a three-day flow diary, applying strengths in daily tasks, and mapping obstacles to engagement, supported by a strength-use tracker and daily reflections on moments of vitality.

Week three, on Relationships, is designed to enhance relational satisfaction through empathy, appreciation, and active-constructive communication. The psychoeducational content emphasizes the role of supportive interactions in improving health and well-being. Participants write and optionally deliver an appreciation letter, practice active-constructive responding, undertake an empathy walk to adopt others’ perspectives, and perform micro-acts of kindness, complemented by daily ratings of social connection and journaling moments of compassion.

In the fourth week, devoted to Meaning, the goal is to help participants connect everyday actions to their personal values and life purpose. The module distinguishes between happiness and meaning and provides strategies for clarifying values. Activities include completing the *Life Compass* to map personal values, daily purpose journaling, writing a legacy letter to the future self, and tracking value-aligned activities, supported by morning intention-setting prompts and evening reflections on value expression.

The fifth and final week, focusing on Accomplishment, aims to develop goal-setting skills, build self-efficacy, and encourage recognition of personal progress. The psychoeducational content outlines the benefits of celebrating incremental achievements. Activities include creating a *Specific, Measurable, Achievable, Relevant, and Time-bound (SMART)* goal plan with milestone breakdowns, recording daily small wins, engaging in the best possible self-visualization, and reflecting on potential obstacles while developing commitment strategies.

##### 2.2.6.2 PPIA+M – App-based intervention plus multiconference group support

Participants assigned to this condition will receive the same five-week mobile intervention as those in the PPIA group, with the addition of a weekly 30-minute telephone-based multiconference session. These sessions will be conducted in small groups (5–6 participants) and facilitated by licensed psychologists or doctoral students trained in group interventions. At the beginning of the first telephone multiconference session, facilitators will explain confidentiality, privacy, and safety rules. Participants will be asked not to disclose information shared by other group members outside the session and may use a preferred first name or pseudonym during calls. They will also be instructed to join the sessions from a private location whenever possible and to use headphones. Each weekly multiconference session will follow a standardized 30-minute structure: a 5-minute check-in, a 10-minute review of completed homework, a 10-minute feedback segment, and a 5-minute wrap-up. Throughout the five sessions, positive or corrective feedback will be delivered in accordance with Miltenberger’s guidelines (51) after reviewing the completed homework. Feedback should be provided immediately after the target behavior and include descriptive praise for correct performance or effort. Corrective feedback should be non-negative, focus on improvement, address one aspect of behavior at a time, and be preceded by praise. Participants will also be encouraged to support one another throughout the change process. Facilitators will be responsible for maintaining time equity, preserving the structured behavioral focus of the session, managing participant interaction around app-based tasks, and ensuring that confidentiality, privacy, and safety rules are followed. If severe distress, suicide risk, trauma-related disclosure, or interpersonal conflict emerges during a call, the facilitator will redirect the group process and activate the Crisis Disclosure Protocol, including private follow-up and referral or emergency procedures when necessary.

##### 2.2.6.3 WLCG – Waitlist Control Group

Participants assigned to the Waitlist Control Group (WLCG) will not receive any intervention during the five-week study period but will complete all study assessments at the same time points as the experimental groups. After completing the final follow-up evaluation, participants in the WLCG will be granted access to the full PPIA mobile intervention program.

#### 2.2.7 Outcome measures

Participants will be assessed at three key time points: pre-intervention (T1), post-intervention (T2), and six-month follow-up (T3), using the instruments listed in **Table 2**. The evaluated variables will include sociodemographic, academic, and clinical characteristics; presence of mental disorders; emotional well-being; psychological, emotional and social well-being; positive emotion; engagement; relationships; meaning; accomplishment; treatment adherence and dropouts; and satisfaction with the intervention, following the same order presented in **Table 2** and **Figure 1**. All evaluations will be conducted in person by independent psychologists blinded to the study’s objectives, hypotheses, intervention conditions, and group assignments. Evaluator training will be provided by two senior psychologists with extensive experience in psychological assessment and intervention studies, consisting of 20 hours of theoretical and practical seminars, including role-playing scenarios and supervised mock assessments to ensure standardized administration of the instrument. A random sample of 10% of the screening interviews will be audio-recorded and independently rated by a second independent blinded psychologist. Interrater agreement for the final eligibility decision (eligible vs. excluded) will be calculated using Cohen’s kappa, with κ ≥.80 considered the minimum acceptable threshold. Any discrepancies will be resolved by consensus with the senior supervisors.

**Table 2:** Variables, measurement instruments, and administration format.

Variable	Measurement instrument	Administration format
Sociodemographic, academic and clinical characteristics	Sociodemographic, academic, and clinical questionnaire	Hetero-administered
Mental disorders	Structured Clinical Interview for DSM-5, Clinician Version (SCID-5-CV; First et al., 2015)	Hetero-administered
Primary outcomes		
Emotional well-being	Warwick–Edinburgh Mental Well-Being Scale (WEMWBS; Tennant et al., 2007)	Self-administered
Psychological, emotional, and social well-being	Mental Health Continuum–Short Form (MHC-SF; Keyes, 2002)	Self-administered
Secondary outcomes		
Positive emotion	Positive and Negative Affect Schedule (PANAS; Watson et al., 1988)	Self-administered
Engagement	Utrecht Work Engagement Scale–Student Version (UWES-S; Schaufeli & Bakker, 2003)	Self-administered
Relationships	Social Connectedness Scale (SCS; Lee & Robbins, 1995)	Self-administered
Meaning	Meaning in Life Questionnaire (MLQ; Steger et al., 2006)	Self-administered
Accomplishment	Achievement Goal Questionnaire–Revised (AGQ-R; Elliot & Murayama, 2008)	Self-administered
Satisfaction with the intervention	Satisfaction with the intervention: Client Satisfaction Questionnaire–8 (CSQ-8; Larsen et al., 1979; Vázquez et al., 2019, Spanish version)	Self-administered
Treatment adherence and dropouts	Recording of the number of modules completed, between-session tasks performed, attendance at the weekly 30-minute multiconference sessions, and dropouts and time	Hetero-administered

SCID-5-CV, Structured Clinical Interview for DSM-5 Disorders, Clinician Version; WEMWBS, Warwick–Edinburgh Mental Well-Being Scale; MHC-SF, Mental Health Continuum–Short Form; PANAS, Positive and Negative Affect Schedule; UWES-S, Utrecht Work Engagement Scale–Student Version; SCS, Social Connectedness Scale; MLQ, Meaning in Life Questionnaire; AGQ-R, Achievement Goal Questionnaire–Revised; CSQ-8, Client Satisfaction Questionnaire–8.

##### 2.2.7.1 Sociodemographic, academic, and clinical characteristics

To collect information on sociodemographic, academic, and clinical variables relevant to participant eligibility, a structured interviewer-administered questionnaire will be used at baseline. It will include information on sex assigned at birth, gender identity, age, marital status, living arrangements, rural/urban setting, employment status, and self-reported socioeconomic level (e.g., monthly household income and access to financial resources). Academic variables will include the current academic program and year of enrollment. Clinical data will include having initiated psychological or psychopharmacological treatment within the two months prior to the study, and currently participating in another mental health–related research study.

##### 2.2.7.2 Presence of mental disorders

For the diagnostic interview, the Structured Clinical Interview for DSM-5-Clinician Version (SCID-5-CV (52)) will be conducted to confirm the presence of any mental disorder, in line with the study’s exclusion criteria. The SCID-5 evaluates a wide range of psychiatric conditions according to DSM-5 criteria, including mood, anxiety, psychotic, dissociative, substance-related disorders, and suicidality, among others. It is considered the gold standard diagnostic tool, with high inter-rater reliability (k = 0.76–0.93).

##### 2.2.7.3 Primary outcome measure: psychological well-being

The primary outcome of the study will be psychological well-being, operationalized through two co-primary measures: global positive mental well-being assessed with the Warwick–Edinburgh Mental Well-Being Scale (WEMWBS (53)) and emotional, psychological and social well-being assessed with the Mental Health Continuum–Short Form (MHC-SF (54)).

The WEMWBS (53) is a 14-item self-report scale designed to measure positive mental well-being as a central component of optimal psychological functioning. Each item is rated on a 5-point Likert scale, ranging from 1 (“none of the time”) to 5 (“all of the time”), producing a total score from 14 to 70, with higher scores reflecting greater well-being. The WEMWBS has demonstrated excellent internal consistency, with Cronbach’s α typically exceeding .88 across samples, and strong test–retest reliability. Validation studies in Spanish university populations confirm its robust psychometric performance, with α values above .85 and expected associations with related constructs of well-being and mental distress.

The MHC-SF (54) is a widely used instrument for assessing emotional, psychological, and social well-being. It consists of 14 items covering positive affect, personal functioning, and social connectedness, each rated on a 6-point scale, ranging from 0 (“never”) to 5 (“every day”). For the present study, the continuous scoring method will be applied, producing scores ranging from 0 to 70, with higher scores indicating greater positive mental health. The MHC-SF has shown excellent reliability, with internal consistency ranging from α = .80 to .89 in community and student populations. Evidence from Spanish validation studies demonstrates high internal consistency (α = .83–.89), strong construct validity, and sensitivity to changes in well-being among university students.

##### 2.2.7.4 Secondary outcomes

###### 2.2.7.4.1 Positive emotion

Positive emotion will be assessed with the Positive and Negative Affect Schedule (PANAS (55)), a 20-item self-administered instrument that evaluates the frequency of positive and negative affective experiences. It consists of two 10-item subscales (positive and negative affect), each rated on a 5-point Likert scale ranging from 1 (“very slightly or not at all”) to 5 (“extremely”). The PANAS has demonstrated high internal consistency (α = .86–.90 for the Positive Affect subscale; α = .84–.87 for the Negative Affect subscale) and good convergent and discriminant validity across clinical and non-clinical populations, including university students.

###### 2.2.7.4.2 Engagement

Engagement will be assessed with the Utrecht Work Engagement Scale–Student Version (UWES-S (56)), a 17-item self-administered instrument that measures vigor, dedication, and absorption. Items are rated on a 7-point Likert scale (0 = “never” to 6 = “always”). The UWES-S has shown good internal consistency (Cronbach’s α ranging from .80 to .90) and factorial validity in student populations.

###### 2.2.7.4.3 Relationships

Relationships will be assessed with the Social Connectedness Scale (SCS (57)), an 8-item self-administered instrument that evaluates the degree of interpersonal closeness and belonging. Items are rated on a 6-point Likert scale (1 = “strongly disagree” to 6 = “strongly agree”), with higher scores reflecting stronger connectedness. The SCS has demonstrated excellent internal consistency (α = .91) and robust construct validity across diverse populations, including students.

###### 2.2.7.4.4 Meaning

Meaning will be assessed with the Meaning in Life Questionnaire (MLQ (58)), a 10-item self-administered instrument with two subscales: presence of meaning and search for meaning. Each item is rated on a 7-point Likert scale (1 = “absolutely untrue” to 7 = “absolutely true”). The MLQ has demonstrated high internal consistency (α = .82–.88) and strong convergent and discriminant validity in both clinical and student samples.

###### 2.2.7.4.5 Accomplishment

Accomplishment will be assessed with the Achievement Goal Questionnaire–Revised (AGQ-R (59)), a 12-item self-administered instrument that evaluates mastery-approach, mastery-avoidance, performance-approach, and performance-avoidance goals as indicators of achievement motivation. Items are rated on a 7-point Likert scale (1 = “not at all true of me” to 7 = “very true of me”). The AGQ-R has shown good internal consistency (α = .77–.90 across subscales) and has been validated in student populations, supporting its use in academic well-being research.

###### 2.2.7.4.6 Satisfaction with the intervention

Satisfaction with the intervention will be assessed at post-intervention (T2) using the Client Satisfaction Questionnaire–8 (CSQ-8 (60); Vázquez et al. (61) for the Castilian Spanish version). The CSQ-8 is an 8-item self-administered instrument rated on a 4-point Likert scale, ranging from 1 to 4, with total scores ranging from 8 to 32, where higher scores indicate greater satisfaction. The original version has demonstrated excellent internal consistency (α >.90), while the Spanish validation confirmed high internal consistency (α = .83) and strong construct validity in clinical and research contexts.

##### 2.2.7.5 Treatment adherence and dropouts

Throughout the study, information on participant dropouts will be systematically recorded, including the time point of withdrawal (T1, T2, or T3) and the stated reasons for discontinuation when available. Treatment adherence will be evaluated by tracking the number of modules completed within the mobile app and the between-session tasks performed by each participant (e.g., experiential exercises, reflections, and homework activities). For participants in the PPIA+M condition, adherence will also include attendance at the weekly 30-minute multiconference sessions. App data logs will automatically capture completion rates and activity engagement, while facilitators will maintain attendance and participation records for the multiconference sessions.

#### 2.2.8 Data management

To protect participant confidentiality, all personal and clinical information will be anonymized through a coding system and stored separately from research data. Physical records will be numbered sequentially and preserved securely for five years following the completion of the study. Collected data will be entered into a secure database designed to exclude any direct personal identifiers. The research team will conduct regular quality control checks, including verification of data ranges and internal consistency, to ensure accuracy throughout the study. Access to both physical files and digital records will be restricted exclusively to authorized research personnel, safeguarded by password-protected systems and secure storage facilities. To prevent data loss, the database will be backed up systematically every 15 days. Finally, all study reports and resulting publications will be prepared in a way that guarantees participant anonymity, ensuring that no identifying information is disclosed.

#### 2.2.9 Statistical analyses

Statistical analyses will be conducted using SPSS version 29.0 and R software (62), following the intention-to-treat (ITT) principle. All randomized participants will be analyzed in the groups to which they were assigned (PPIA, PPIA+M, WLCG), regardless of intervention adherence or dropout.

Missing data will be handled using multiple imputation by chained equations (MICE).

The imputation model will include the following predictors: sociodemographic characteristics (sex, gender identity, age, marital status, living arrangements, rural/urban setting, employment status, and self-reported socioeconomic level), academic variables (current academic program, year of enrolment), clinical characteristics (having initiated psychological or psychopharmacological treatment within the two months prior to the study, currently participating in another mental health–related research study), and baseline scores on the primary outcomes (emotional well-being and psychological, emotional, and social well-being).

To analyze the effect of the intervention on the primary outcome, psychological well-being, operationalized through two co-primary measures—global positive mental well-being assessed with the WEMWBS and emotional, psychological, and social well-being assessed with the MHC-SF—separate Linear Mixed Models (LMMs) will be conducted for each co-primary outcome using Restricted Maximum Likelihood (REML) estimation, with fixed effects for group, time, and the group-by-time interaction. Time will be treated as a categorical factor with three levels (T1: baseline, T2: post-intervention, T3: six-month follow-up), and random intercepts will be included to account for intra-subject variability. The baseline assessment (T1) will be modeled as part of the repeated outcome vector rather than as a separate baseline covariate. Random slopes for time will not be included in the primary models.

Model assumptions, including the normality and homoscedasticity of residuals, will be evaluated using Q-Q plots and residual-versus-predicted scatterplots before final model interpretation.

To control the family-wise Type I error rate across the two co-primary outcomes, a Bonferroni-adjusted significance threshold of α = .025 will be applied to the primary confirmatory group-by-time interaction test for each outcome. *Post hoc* pairwise comparisons among the three groups (PPIA vs. WLCG, PPIA+M vs. WLCG, and PPIA+M vs. PPIA) will be conducted using estimated marginal means at post-intervention (T2) and six-month follow-up (T3). Pairwise contrasts will be adjusted using the Bonferroni correction. Effect sizes will be calculated using Cohen’s *d* and interpreted as small (*d* < 0.50), moderate (*d* = 0.50–0.79), or large (*d* ≥ 0.80), following the guidelines proposed by Cohen (63).

##### 2.2.9.1 Clinical significance

As a complementary analysis, reliable change will be examined using the Jacobson and Truax approach (64). The Reliable Change Index will be calculated as RCI = (post-intervention score - baseline score)/SEdiff. Participants with RCI values greater than 1.96 will be classified as showing positive reliable change, values below -1.96 as showing negative reliable change, and values between -1.96 and 1.96 as showing no reliable change. If appropriate normative reference values are available for the WEMWBS and MHC-SF in comparable student populations, clinical significance cutoffs will also be calculated; otherwise, interpretation will focus on reliable change rather than clinical recovery.

##### 2.2.9.2 Moderation and mediation analyses

Moderation and mediation analyses will be conducted as exploratory and hypothesis-generating analyses for the PPIA, PPIA+M, and WLCG groups. These analyses are not part of the primary confirmatory testing framework and will not be interpreted as demonstrating causal mechanisms.

Moderation analyses will be conducted using regression models including treatment condition, the candidate moderator, and the treatment-by-moderator interaction term. Continuous variables will be mean-centered to improve interpretation, following the recommendations of Kraemer and Blasey (65). Potential moderators will include baseline sociodemographic, academic, and clinical variables, adherence, and satisfaction with the intervention. Evidence of moderation will be considered present when the treatment-by-moderator interaction term is statistically significant, indicating that the effect of the intervention on well-being outcomes varies according to the level of the moderator.

Mediation analyses will examine whether changes in PERMA dimensions —positive emotion, engagement, relationships, meaning, and accomplishment— are associated with changes in emotional well-being and psychological, emotional, and social well-being. Changes in WEMWBS and MHC-SF scores between baseline and post-intervention will be used as dependent variables, treatment condition as the independent variable, and changes in PERMA dimensions between baseline and post-intervention as potential mediators. Indirect effects will be estimated using bias-corrected bootstrap confidence intervals with 5,000 resamples. Because mediator and outcome changes are assessed over overlapping time intervals, these analyses will be considered exploratory and will not be interpreted as demonstrating causal mechanisms.

#### 2.2.10 Exit strategy

An exit strategy will be implemented in two key scenarios: (1) when a participant decides to withdraw from the study prematurely, and (2) at the official conclusion of the trial. In cases of premature withdrawal, a voluntary exit phone call will be conducted to document the reasons for leaving through a structured questionnaire. At the end of the study period, which includes a five-week intervention period followed by a six-month follow-up, participants will be informed about the transition and closure of the research.

## 3 Discussion

This study will evaluate the efficacy of a brief positive psychology–based psychological intervention grounded in the PERMA model, delivered via a mobile application (PPIA) and through a blended format incorporating weekly multiconference telephone sessions (PPIA+M). The target population consists of university students, who face unique developmental, academic, and social stressors that increase their vulnerability to distress. Grounded in previous research on positive psychology and digital interventions (20, 24, 26, 28), the intervention systematically incorporates the five PERMA domains—Positive Emotion, Engagement, Relationships, Meaning, and Accomplishment—into a structured digital program. In line with evidence on telephone-based psychosocial support (30, 32–34), we hypothesize that both active intervention groups will outperform the waitlist control, with PPIA+M yielding higher adherence and impact due to added human contact.

The design of this intervention aligns with established best practices in prevention and mental health promotion among students, incorporating evidence-based elements positive psychology and cognitive-behavioral interventions while addressing the known barriers to access and engagement (25, 27). The PERMA framework (20) provides theoretical consistency and breadth, as it addresses psychological functioning beyond symptom reduction. This multidimensional focus makes it particularly suitable for prevention in nonclinical populations such as university students.

This study also proposes the use of ICTs to enhance accessibility and engagement. The mobile app enables flexible, autonomous completion of psychoeducational and experiential tasks, offering accessibility, scalability, and cost-efficiency. The PPIA+M condition introduces synchronous group contact via weekly multiconference calls. This blended approach is expected to maximize engagement, adherence, and therapeutic outcomes (30, 35) by enhancing motivation, strengthening therapeutic alliance, and fostering community (33).

The methodological rigor of this study is reinforced by adherence to updated SPIRIT 2025 (37) recommendations for clinical trial protocols and was informed by CONSORT 2025 (36) guidelines where applicable. Specifically, the protocol complies with SPIRIT recommendations regarding trial registration, specification of eligibility criteria, detailed description of interventions, outcome specification with validated instruments, sample size determination, randomization procedures, blinding of outcome assessment, and statistical analysis methods. In line with CONSORT, the study ensures a comprehensive description and manualization of interventions, training and supervision of facilitators, monitoring of fidelity, specification of comparator groups, use of psychometrically robust outcome measures, reporting of adherence and attrition, and inclusion of mediation and moderation analyses to clarify mechanisms of change. The intervention is fully manualized, facilitators receive training with fidelity checks, and blinded evaluators will use validated instruments. The primary outcomes—emotional well-being and psychological, emotional, and social well-being—will be assessed using the Warwick–Edinburgh Mental Well-Being Scale (WEMWBS) and the Mental Health Continuum–Short Form (MHC-SF).

However, some limitations should be acknowledged. First, the sample will be drawn from a single university, which may limit the generalizability of the findings to other academic or cultural contexts, and participation will be voluntary, raising the possibility of self-selection bias. Voluntary enrollment may attract students with higher baseline motivation, greater interest in psychological well-being, or greater readiness to engage in self-guided activities. Second, outcomes will rely primarily on self-report measures, which may be subject to social desirability bias, reporting biases, and measurement fatigue across repeated assessments. Third, the use of a waiting list control group, while ethically and practically justified, may lead to an overestimation of intervention effects compared with active control conditions. Moreover, this passive comparator does not allow the specific active ingredients of the PERMA model to be isolated from non-specific factors such as digital novelty, participant expectancy, structured self-monitoring, or human support. Therefore, any efficacy findings should be interpreted as supporting the effectiveness of the intervention package relative to no immediate intervention, rather than demonstrating the specific incremental value of the PERMA components alone. Fourth, adherence is expected to play a critical role in determining intervention effectiveness, particularly in self-guided digital formats. Because telephone multiconference support will be delivered in small groups and facilitated by trained psychologists, minor peer-group or facilitator effects cannot be completely ruled out. Although these effects are expected to be limited because the sessions are brief, manualized, and focused on adherence feedback rather than group psychotherapy, they should nevertheless be considered when interpreting differences between PPIA and PPIA+M. Future studies should replicate these findings using multisite samples, active comparators, and complementary assessment strategies.

In conclusion, this research is innovative in testing a brief, theoretically grounded PERMA-based intervention through digital and hybrid delivery. If efficacious, it could be adopted across academic contexts as a scalable, cost-effective, and engaging tool to promote student mental health. By addressing methodological gaps, integrating technology with human support, and focusing on resilience and academic functioning, this study could inform future interventions for young adults and generate impact across academic, clinical, and social domains.
